# P-1915. The Giant Egg Challenge: Leveraging Peer-Led Education to Teach Vaccine-Preventable Diseases to College Students

**DOI:** 10.1093/ofid/ofaf695.2084

**Published:** 2026-01-11

**Authors:** Sheri Leung, Britney Phung, Yvonne Chen, Nicole Bradley, Yuman Lee

**Affiliations:** St. John's University, Fresh Meadows, NY; St. John's University, Fresh Meadows, NY; St. John's University, Fresh Meadows, NY; St. John's University, Fresh Meadows, NY; St. John's University, Fresh Meadows, NY

## Abstract

**Background:**

College students are at increased risk for vaccine-preventable diseases (VPDs) due to close living quarters, frequent social interactions, international travel, and inconsistent access to healthcare. Improving their awareness of VPDs is critical to reducing vaccine hesitancy, maintaining herd immunity, and avoiding delayed response during outbreaks. Peer-led education and game-based learning are effective strategies for engaging this population. Under the mentorship of two faculty members, three pharmacy students developed *The Giant Egg Challenge: Vaccines Edition*, a peer-led, TikTok-inspired trivia game where players answer questions about VPDs to crack the most eggs and raise awareness. This study aimed to evaluate the impact of this activity on college students’ perceived knowledge and attitudes of VPDs.

**Methods:**

Pharmacy students led *The Giant Egg Challenge: Vaccines Edition* at a university-wide health fair in April 2025 to educate peers about VPDs. Participants completed an anonymous survey with four Likert-scale questions to assess the activity’s impact on their perceived knowledge and attitudes toward VPDs.

**Results:**

Out of 86 responses, the majority (97.7%) agreed or strongly agreed that the activity made learning about VPDs more engaging. Additionally, 94.2% agreed or strongly agreed that they felt more confident in their knowledge of VPDs and that they learned new information during the activity. Furthermore, 96.5% agreed or strongly agreed that vaccines play an important role in protecting public health.

**Conclusion:**

This study demonstrates the effectiveness of peer-led, game-based education in raising awareness of VPDs among college students. By leveraging peer-led education and a viral TikTok trend, *The Giant Egg Challenge: Vaccines Edition* provided an engaging, relatable learning experience. College students are a critical target for vaccine education, as a survey at the same health fair found that 11–13% self-reported being unvaccinated or unsure of their vaccination status for mandated vaccines such as MMR and meningococcal meningitis. These gaps threaten herd immunity and delay outbreak responses. This creative approach effectively combined peer influence, social media trends, and gameplay to deliver meaningful public health education.

**Disclosures:**

All Authors: No reported disclosures

